# Correlation Analysis of Ultrasonic Pulse Velocity and Mechanical Properties of Normal Aggregate and Lightweight Aggregate Concretes in 30–60 MPa Range

**DOI:** 10.3390/ma15082952

**Published:** 2022-04-18

**Authors:** Wonchang Kim, Keesin Jeong, Hyeonggil Choi, Taegyu Lee

**Affiliations:** 1 Department of Fire and Disaster Prevention, Semyung University, Choongbuk 27136, Korea; kimwc69082@gmail.com (W.K.); jks@semyung.ac.kr (K.J.); 2 School of Architecture and Civil Engineering, Kyungpook National University, Daegu 41566, Korea

**Keywords:** normal aggregate concrete, lightweight aggregate concrete, ultrasonic pulse velocity, strength prediction, elastic modulus prediction model, compressive strength, elastic modulus

## Abstract

This study classified the strength of normal aggregate concrete (NC) and lightweight aggregate concrete (LC) into three levels (30, 45, and 60 MPa). In particular, the compressive strength, ultrasonic pulse velocity, and elastic modulus were measured and analyzed at the ages of 1, 3, 7, and 28 days to establish the correlation between the compressive strength and the ultrasonic pulse velocity and between the elastic modulus and the ultrasonic pulse velocity. In addition, this study proposed strength and elastic modulus prediction equations as functions of the ultrasonic pulse velocity. The developed equations were compared with previously proposed strength prediction equations. The results showed that the measured mechanical properties of NC tended to be higher at all ages than in LC. However, LC45 exhibited relatively high compressive strength compared to NC45. The relative mechanical properties of LC compared to NC were the highest at 45 MPa and the lowest at 60 MPa. The relative ultrasonic pulse velocity converged at all levels as the age increased. Moreover, the correlation between the compressive strength and the ultrasonic pulse velocity in LC exceeded that of NC, and in LC, the correlation coefficient decreased as the strength increased. The correlation coefficients between the elastic modulus and the ultrasonic pulse velocity were high at all levels except for LC45. Finally, this study proposed compressive strength and elastic modulus prediction equations as an exponential function of LC. The proposed equations outperformed the previously proposed strength prediction equations.

## 1. Introduction

As the demand for large-scale and high-rise building construction increases, the amount of cement and aggregates mixed into these buildings increases. In general, cement and normal aggregate have densities of 3140 kg/m^3^ and 2240–2400 kg/m^3^, respectively. Thus, as the amount of cement and aggregates mixed increases, the self-weight of the building and the load increase accordingly [1,2,3,4]. Moreover, creep deformation under long-term and continuous load adversely affects the structure’s durability. This also has adverse economic effects, such as an increase in the materials used due to a larger cross-sectional area of the compression members supporting self-weight, or reduced space usability [5].

To address the problems mentioned above, researchers have studied methods to reduce the self-weight of buildings by mixing lightweight aggregate instead of normal aggregate [6,7]. Compared to normal aggregate concrete (NC), lightweight aggregate concrete (LC) generally has superior seismic resistance, due to reduced self-weight and excellent soundproofing, thermal insulation, and fire resistance owing to its numerous voids. ACI (American Concrete Institute) and ASTM in the US and JASS in Japan specify the standards for structural LC. According to ACI 211.2 & 213.R, the compressive strength of a mixture of coarse and fine aggregates as a lightweight aggregate is 17–28 MPa. Moreover, the compressive strength of a mixture of only fine aggregates as a lightweight aggregate is 17–28 MPa. In addition, a damage-ratio strength criterion was proposed by Ding et al. [8], relying on a six-parameter expression of the damage ratio variable and considering the angle of Lode and the hydrostatic pressure on NC and LC. The proposed expression was compared to previous criteria.

However, as LC has numerous voids, this material has high absorption and drying shrinkage, which promote carbonation and reduce strength [9,10,11]. Therefore, standards for strength range and strength prediction when using structural LC were developed. For instance, ASTM C330 specifies compressive strength of at least 17.5–19 MPa and ultrasonic pulse velocity of at least 2.18–2.76 km/s. In contrast, JASS 5 specifies compressive strength of up to 27–36 MPa [12,13,14,15,16].

The strength of structural concrete is a crucial mechanical property. In particular, emphasis on predicting the strength of LC to compare it with that of NC is essential. In this regard, strength can be predicted using ultrasonic pulse velocity, which is a non-destructive test often applied in the field. Researchers have actively studied strength prediction techniques using ultrasonic pulse velocity. The ultrasonic pulse velocity is affected by the concrete aggregate’s density and shape, the interface between the aggregate and paste, and the number of voids. Previous studies reported that owing to the low density and porosity of LC, the ultrasonic pulse velocity of LC tends to be lower than that of NC.

Furthermore, Shafigh et al. [17] studied LC by mixing fly ash (FA), limestone powder, and oil palm shell (OPS). This obtained a strength in the range of 5.8–41.5 MPa and an ultrasonic pulse velocity of 3.71–3.89 km/s. The authors proposed a strength prediction model with a correlation coefficient (R^2^) of 0.94 based on an analysis of ultrasonic pulse velocity. Nikbin et al. [18] studied LC by mixing red mud and expanded clay fine aggregate. The authors reported strength in the range of 18.83–28.83 MPa, an ultrasonic pulse velocity of 3.7–4.3 km/s, and an elastic modulus in the range of 14–18 GPa. In addition, the authors proposed a strength prediction model with R^2^ = 0.84. Majhi et al. [19] studied LC by replacing normal aggregate with sintered FA. The authors reported a strength in the range of 11.3–35.3 MPa and an ultrasonic pulse velocity of 3.2–5 km/s. The study also proposed a strength prediction model with R^2^ = 0.96. Similarly, Akcaozoglu et al. [20] evaluated LC using waste PET to replace the coarse aggregate. The authors obtained a strength in the range of 8.4–26.9 MPa and an ultrasonic pulse velocity of 2.32–2.46 km/s. They proposed a strength prediction model with R^2^ = 0.93.

Previous studies have only investigated trends in ultrasonic pulse velocity according to the age of LC or the substitution rate of admixtures and aggregates. Thus, research considering ultrasonic pulse velocity according to the strength of LC remains to be undertaken. In addition, most previous studies that proposed a prediction model analyzed the correlation between the compressive strength and the ultrasonic pulse velocity, with little focus on predicting the elastic modulus through ultrasonic pulse velocity. Furthermore, few studies have measured the ultrasonic pulse velocity of LC at compressive strengths of 40 MPa or more. Thus, strength prediction equations based on ultrasonic pulse velocity in LC of 40 MPa or more might have reduced accuracy. 

**Figure 1:**
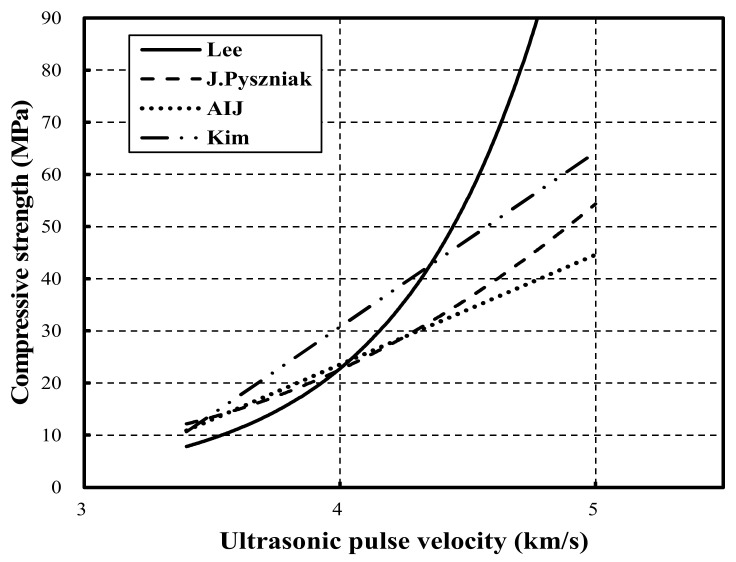
Previous studied relationship between compressive strength and ultrasonic pulse velocity [21,22,23,24].

Accordingly, this study analyzed the ultrasonic pulse velocity and other mechanical properties of LC and NC at different strengths. The strengths for NC and LC were set to 30, 45, and 60 MPa. Regarding the mechanical properties, the compressive strength, elastic modulus, and ultrasonic pulse velocity were measured. In particular, compressive strength and ultrasonic pulse velocity were measured at 1, 3, 7, and 28 days, while elastic modulus was measured at 3, 7, and 28 days. The reason for these settings was to analyze how the type of mixed aggregate of NC and LC affected the mechanical properties at different strengths, and to compare the differences to understand the characteristics of these materials. This study also analyzed the correlation between compressive strength and ultrasonic pulse velocity in NC and LC and the correlation between elastic modulus and ultrasonic pulse velocity according to strength. Through this analysis, the strength range can be identified to accurately predict compressive strength and elasticity coefficient by ultrasonic pulse velocity analysis. The analysis also identifies the strength range requiring further research. Finally, prediction equations for the elastic modulus and strength of LC are proposed and compared with previous strength prediction equations (Table 1, Figure 1) [21,22,23,24].

## 2. Experimental Procedure

### 2.1. Materials

Table 2 shows the physical properties of cement, coarse aggregate, and fine aggregate. This study used Type I Portland Cement (density: 3150 kg/m^3^ and fineness: 320 m^2^/kg). For the coarse aggregate, crushed granite aggregate (density: 2680 kg/m^3^, fineness modulus: 7.03, absorption: 0.68%, and maximum size: 20 mm) and coal ash aggregate (density: 1470 kg/m^3^, fineness modulus: 6.39, absorption: 8.68%, and maximum size: 20 mm) were used. For the fine aggregate, river sand (density: 2540 kg/m^3^, fineness modulus: 2.54, and absorption: 1.6%) was used. For the admixture, high-performance polycarboxylic-based acid water-reducing admixture was used. Table 3 shows the chemical properties of cement.

### 2.2. Experimental Program and Concrete Mix Proportion

Table 4 shows the experimental program. Crushed granite aggregate was mixed with NC and coal ash aggregate was mixed with LC [25]. For both concrete types, the target strengths were set at 30, 45, and 60 MPa. They were cured at constant temperature and humidity until 1 day of age; the mixture was then demolded and cured in water until 28 days. Compressive strength (MPa), ultrasonic pulse velocity (km/s), and elastic modulus (GPa) were measured.

Table 5 shows the concrete mix proportions used in this study. The mixture was divided into NC and LC based on the same target strengths. For the target strengths, water/binder (W/B) was set to 41.3%, 33.0% and 28.0%.

### 2.3. Test Methods

Table 6 shows the test items for the mechanical properties, standards, and ages. The compressive strength tests were performed at 1, 3, 7, and 28 days according to ASTM C39/C39M [26]. The ultrasonic pulse velocity tests were conducted at 1, 3, 7, and 28 days according to ATM C597 [27]. The elastic modulus tests were conducted at 3, 7, and 28 days according to ASTM C469 [28]. The ultrasonic pulse velocity of concrete was calculated using Equation (1):

(1) $$Vp=\frac{L}{\Delta t},$$

where *Vp* is the ultrasonic pulse velocity (km/s), *L* is the distance (km), and △*t* is the transit time (s).

## 3. Results and Discussion

### 3.1. Mechanical Properties of NC and LC

**Figure 2:**
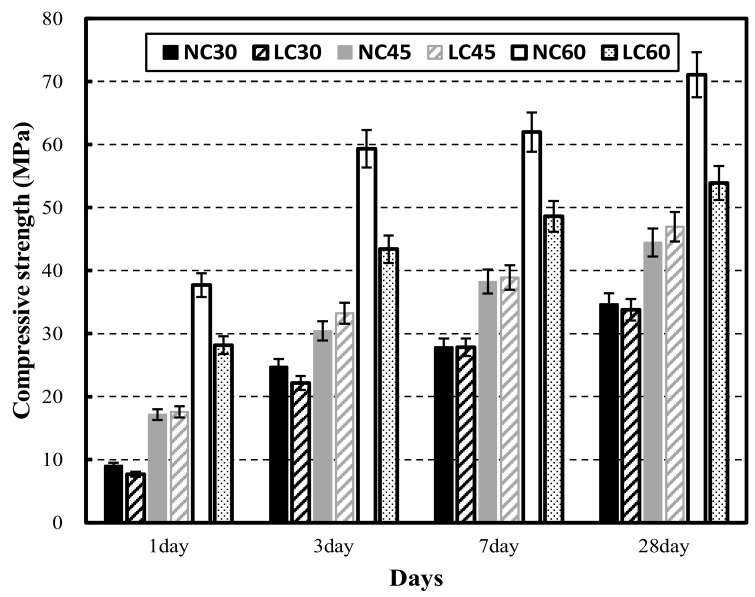
Compressive strength of normal aggregate concrete and lightweight aggregate concrete at different ages.

**Figure 3:**
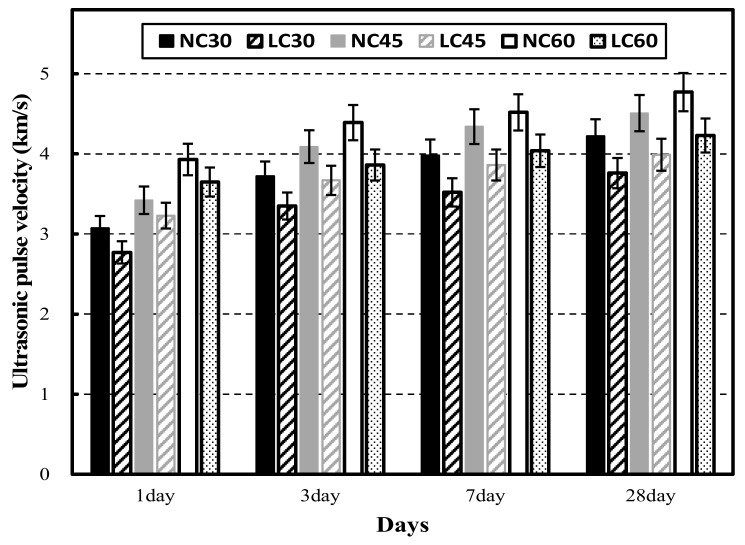
Ultrasonic pulse velocity of normal aggregate concrete and lightweight aggregate concrete at different ages.

**Figure 4:**
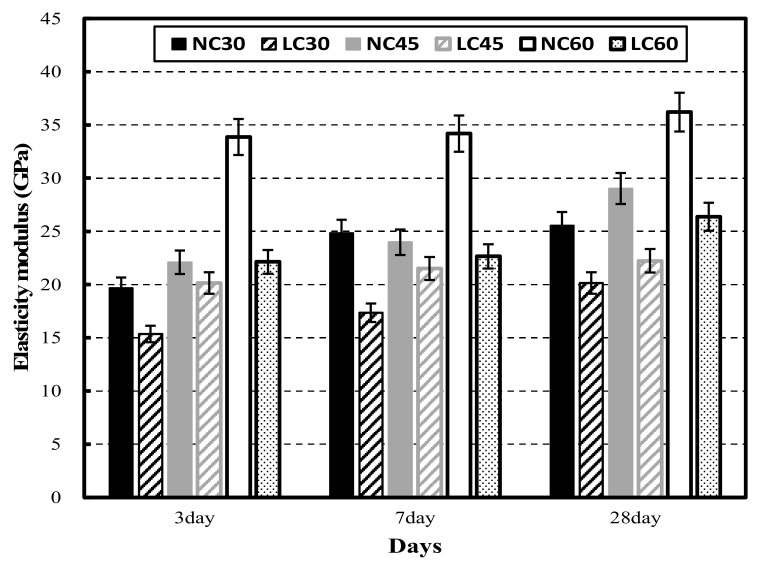
Elastic modulus of normal aggregate concrete and lightweight aggregate concrete at different ages.

The mechanical properties of NC and LC are shown by age in Figure 2, Figure 3 and Figure 4, from left to right, in the following order: NC30, LC30, NC45, LC45, NC60, and LC60. In particular, Figure 2 shows the compressive strengths of NC and LC at 1, 3, 7, and 28 days of age. Both NC30 and LC30 developed the target strength. Although NC30 developed slightly higher strength than LC30, overall, the strengths were similar. NC45 did not develop the target strength, while LC45 did develop the target strength. LC45 exhibited higher strength than NC45 at all ages. The reason might be that the moisture in the lightweight aggregate promoted the hydration reaction of cement, further developing the strength. NC60 developed the target strength, whereas that of LC60 was somewhat lower than NC60.

Figure 3 shows the ultrasonic pulse velocities of NC and LC at 1, 3, 7, and 28 days of age. Note that as age increases, the ultrasonic pulse velocity increases. The ultrasonic pulse velocity of NC exceeded that of LC at all ages. The reason might be that the ultrasonic pulse velocity was greatly impacted by the coarse aggregate and was more affected by the low density and porosity of the lightweight aggregate compared to granite aggregate [29,30,31]. At 1 day of age, the ultrasonic pulse velocity was at least 3 km/s in all cases except LC30. The ultrasonic pulse velocity of NC was at least 4 km/s at 7 days of age, and at 28 days, the ultrasonic pulse velocity was at least 4 km/s at all levels except LC30.

Figure 4 shows the elastic modulus of NC and LC at 3, 7, and 28 days of age. NC exhibited a higher elastic modulus than LC at all ages. At 28 days, the elastic modulus of NC at all levels was at least 25 GPa, while that of all LC levels except for LC60 was approximately 20 GPa.

### 3.2. Relative Mechanical Properties between NC and LC

**Figure 5:**
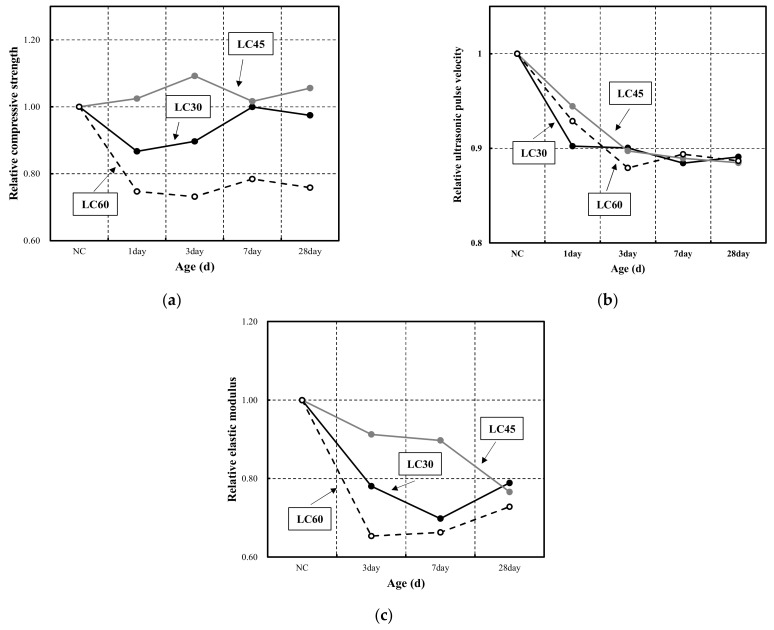
Relative mechanical properties between normal aggregate concrete and lightweight aggregate concrete at different ages: (**a**) relative compressive strength; (**b**) relative ultrasonic pulse velocity; (**c**) Relative elastic modulus.

Figure 5 shows the relative mechanical properties of the LC compared to NC at the same strength levels at different ages. In particular, Figure 5a shows the relative compressive strength by age. The greatest difference between LC30 and NC30 was observed at 1 day of age, and the difference decreased as the age increased. After 7 days, the strength was nearly identical to that of NC. A larger difference was noted in the relative compressive strength between LC60 and NC60, with the largest difference observed at 3 days. The difference was smallest at 7 days at approximately 78%. LC45 exhibited higher compressive strength than NC45; the largest difference in compressive strength was observed at 3 days.

Figure 5b shows the relative ultrasonic pulse velocities at different ages. In the tests, LC showed relatively low ultrasonic pulse velocities compared to NC at all levels, and the difference from NC grew as the age increased. At 1 day of age, the difference in the ultrasonic pulse velocity of LC45 was the smallest, and the difference in that of LC30 was the largest. At 3 days, LC30 and LC45 were similar, whereas LC60 showed the largest difference. However, at later ages, the three levels exhibited similar trends.

Figure 5c shows the relative elastic modulus according to age. In particular, LC presented a lower elastic modulus than NC at all levels. Until 7 days of age, the relative elastic modulus of LC45 was the smallest, and at 28 days, LC30 exceeded LC45. For LC30, the difference in elastic modulus increased until 7 days of age, and the difference decreased at 28 days. For LC60, the difference in elastic modulus increased until 3 days of age and gradually decreased as age increased. For LC45, the difference in elastic modulus continued to increase as age increased. LC30 and LC60 exhibited similar trends by age; however, they presented the largest difference of approximately 0.13 at 3 days, and the difference was reduced to 0.04 and 0.06 at 7 and 28 days.

### 3.3. Comparison between Compressive Strength and Ultrasonic Pulse Velocity

**Figure 6:**
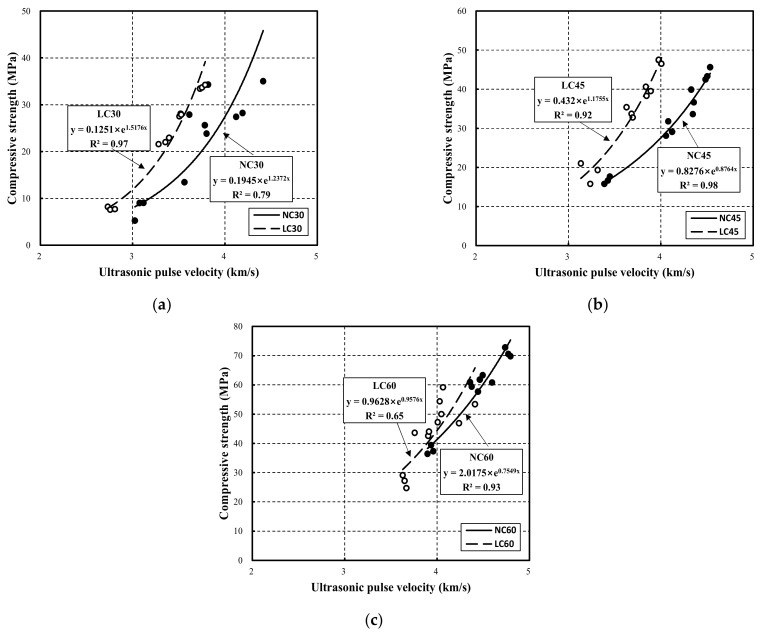
Comparison correlationship between compressive strength and ultrasonic pulse velocity in normal aggregate concrete and lightweight aggregate concrete: (**a**) 30 MPa; (**b**) 45 MPa; (**c**) 60 MPa.

Figure 6 compares compressive strength and ultrasonic pulse velocity for NC and LC.

As shown in the figure, the relationship between the compressive strength and the ultrasonic pulse velocity according to the strength of NC and LC follows an exponential function. Here, this function is expressed in Equation (2). Moreover, the results of the proposed equation were analyzed and compared with those obtained in existing studies [32,33,34,35,36,37,38,39].

(2) $$ƒc=A\cdot e^{\;B*Vp},$$

where ƒc is the compressive strength, *A* and *B* are empirical constants, and *Vp* is the ultrasonic pulse velocity.

NC30 exhibited a low R^2^ of 0.79, whereas NC45 and NC60 exhibited relatively high values of 0.98 and 0.93. Moreover, LC30, LC45, and LC60 presented R^2^ of 0.97, 0.92, and 0.65, respectively, indicating that R^2^ decreased as the strength increased. The reason for this is that in the compressive strength range of 50–60 MPa, where the microstructure changes, the ultrasonic pulse velocity is not governed by the strength, due to changes in surface stiffness and micro-voids. Instead, it is affected by the hydration product structure, aggregate strength, and factors affecting the measurements, such as numerous voids on the matrix and structural fixation, owing to the rapid initial hardening [40,41].

The compressive strength and ultrasonic pulse velocity correlation graph showed that LC exceeded NC at all strengths, while LC60 and NC60 exhibited similar trends. The correlation equations and the corresponding R^2^ are listed in Table 7.

### 3.4. Comparison between Elastic Modulus and Ultrasonic Pulse Velocity

**Figure 7:**
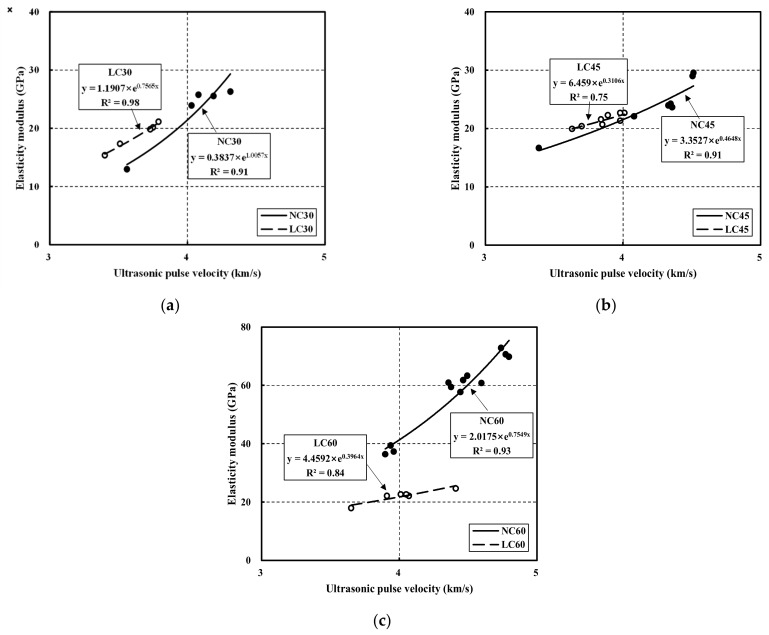
Relationship between elastic modulus and ultrasonic pulse velocity in normal aggregate concrete and lightweight aggregate concrete: (**a**) 30 MPa; (**b**) 45 MPa; (**c**) 60 MPa.

Figure 7 compares elastic modulus and ultrasonic pulse velocity for NC and LC. In particular, NC30 and LC30 exhibited R^2^ of 0.91 and 0.98, respectively. Therefore, the elastic modulus and ultrasonic pulse velocity strongly correlated at 30 MPa. Moreover, NC45 and LC45 showed an R^2^ of 0.91 and 0.75, respectively. 

Although NC showed a higher correlation at 45 MPa than at 30 MPa, LC exhibited a relatively low correlation. NC60 and LC60 had R^2^ of 0.93 and 0.84; hence, NC showed a strong correlation at 60 MPa, whereas LC showed a relatively low correlation. Overall, the elastic modulus might be predicted through the ultrasonic pulse velocity [42,43,44]. 

Nevertheless, further research on strengthening the relationship between the ultrasonic pulse velocity and the elastic modulus is required. The correlation equations with the corresponding R^2^ are listed in Table 8.

### 3.5. Proposed Prediction Equations of Compressive Strength and Elastic Modulus by Ultrasonic Pulse Velocity

**Figure 8:**
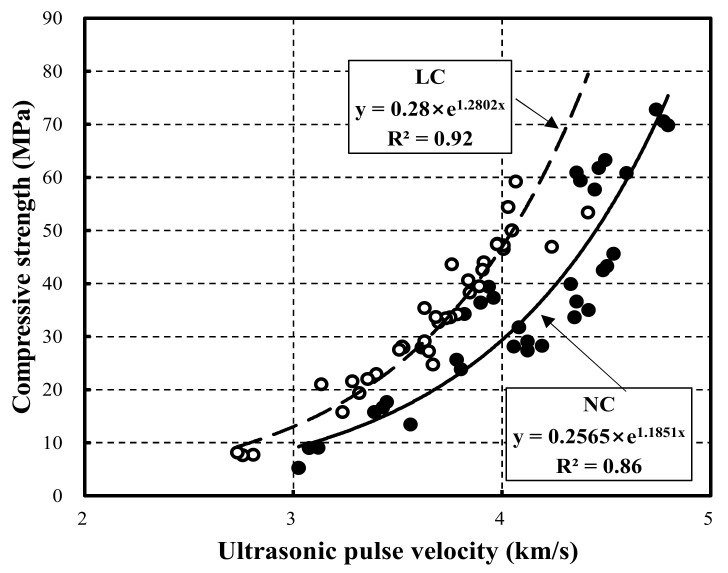
Relationship between compressive strength and ultrasonic pulse velocity in the estimation equations.

Figure 8 shows the relationship between the compressive strength and the ultrasonic pulse velocity using previously proposed estimation equations [32,33,34,35,36,37,38,39]. Overall, the relationship follows an exponential function. In particular, LC and NC exhibited R^2^ of 0.92 and 0.86, respectively. Hence, the R^2^ of LC was higher than that of NC. Moreover, the curve for LC exceeded that of NC. 

**Figure 9:**
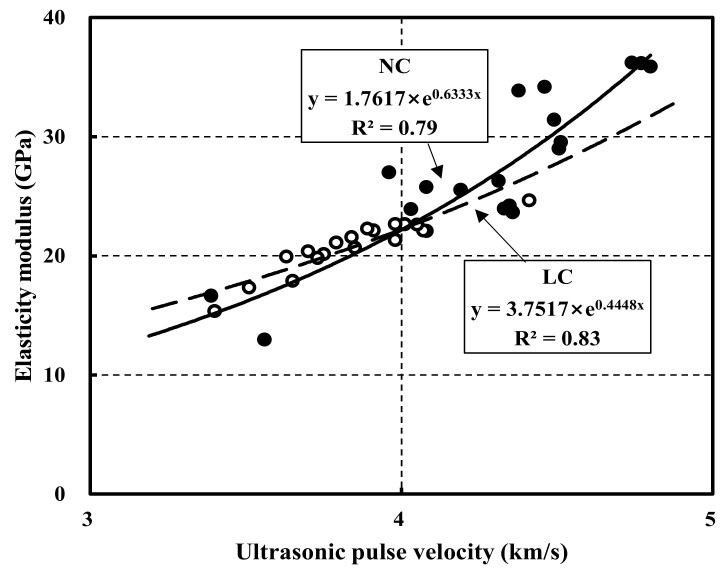
Relationship between elastic modulus and ultrasonic pulse velocity in estimation equation.

Figure 9 shows the relationship between the elastic modulus and the ultrasonic pulse velocity using the estimation equations proposed in [42,43,44]. LC and NC exhibited R^2^ of 0.83 and 0.79, respectively. Thus, the R^2^ of LC was higher than that of NC. Furthermore, in the range under 4 km/s, the curve of LC exceeded that of NC, whereas in the subsequent range, the curve of NC exceeded that of LC. However, the R^2^ was observed be lower than that between the compressive strength and the ultrasonic pulse velocity. Therefore, further research is required to propose estimation equations that strongly correlate the elastic modulus and the ultrasonic pulse velocity.

**Figure 10:**
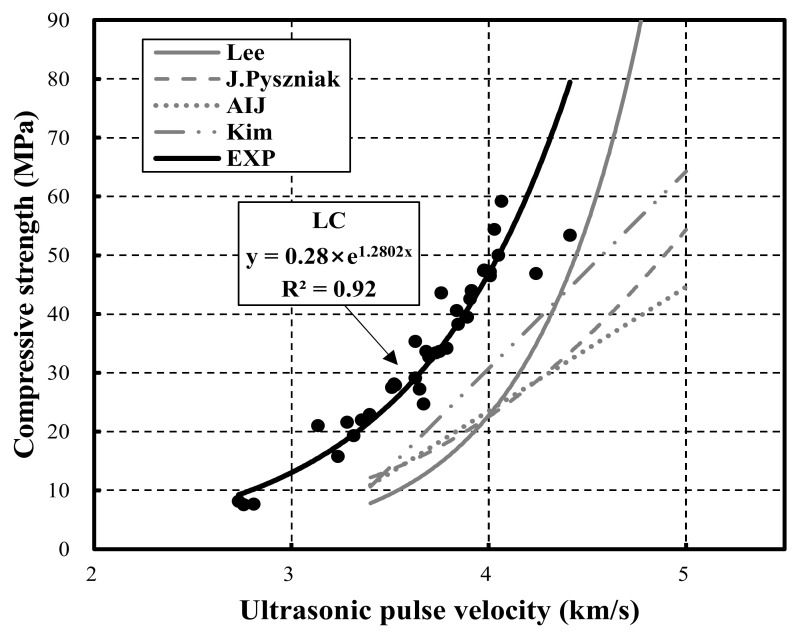
Comparison between previous prediction equations and the herein-proposed estimation equation.

Figure 10 compares the previous strength prediction equations and the LC strength prediction equation proposed in this study. The previous strength prediction equations exhibit a similar trend in the range of approximately 3.3–3.4 km/s.

In contrast, the LC strength prediction equation proposed in this study outperformed the previous strength prediction equations [21,22,23,24]. This result indicates that errors occur in the prediction range of compressive strength and elastic modulus for materials that have undergone a different development process in the production of lightweight aggregate. Furthermore, compared to existing equations, the proposed strength prediction model can be used to predict the strength of LC using coal-ash-based lightweight aggregate accurately.

## 4. Conclusions

This study classified NC and LC by strength (30, 45, and 60 MPa). The compressive strength, ultrasonic pulse velocity, and elastic modulus according to the age of the tested samples were measured and analyzed. The results are summarized below.

(1) NC30 and LC30 showed similar strength development by age. Notably, LC45 exhibited higher strength development than NC45. The reason for this was that the moisture in the lightweight aggregate promoted the hydration reaction of cement, further developing the strength. In contrast, LC60 showed minimal strength development compared to NC60. The ultrasonic pulse velocity tended to increase as the age increased. Moreover, the ultrasonic pulse velocity of LC was lower than that of NC at the same strength. These differences might be related to the density of the mixed coarse aggregates. Based on the same strength, the elastic modulus of NC was higher than that of LC, and the elastic moduli of NC60 and LC60 differed the most.

(2) Regarding the relative compressive strength of LC compared to NC, LC45 showed a higher compressive strength than NC45 at all ages. However, LC30 and LC60 exhibited relatively low compressive strength compared to NC. The relative ultrasonic pulse velocity of LC compared to NC showed a similar trend as the age increased. The relative elastic modulus of LC showed the slightest difference from NC in LC45 and the largest difference in LC60 up until 7 days of age. Moreover, the difference was small at 28 days.

(3) The correlation between the compressive strength and the ultrasonic pulse velocity of NC30, NC45, and NC60 were 0.79, 0.98, and 0.93, respectively. In addition, the correlation for LC30, LC45, and LC60 were 0.97, 0.92, and 0.65, respectively. For LC, the correlation decreased as the strength increased. Note that at all strengths, the curves of LC tended to exceed those of NC.

(4) The correlation between the elastic modulus and the ultrasonic pulse velocity of NC30, NC45, and NC60 were 0.91, 0.91, and 0.93, respectively, showing a strong correlation. The correlations for LC30, LC45, and LC60 were 0.98, 0.75, and 0.84, respectively, exhibiting, in general, a lower correlation than NC. Except for NC60 and LC60, the curves of LC at the same target strength exceeded those of NC.

(5) This study proposed prediction equations for strength and the elastic modulus based on the ultrasonic pulse velocity. For NC and LC, the strength prediction equations exhibited a correlation of 0.86 and 0.92, respectively. Moreover, the elasticity modulus prediction equations exhibited correlations of 0.79 and 0.83.

Within the scope of this study, we proposed prediction equations based on the relationships between the ultrasonic pulse velocity and mechanical properties such as compressive strength and elastic modulus according to the aggregate type. The findings demonstrated that factors such as the composition and size of aggregates have a clear tendency to exceed the range of existing prediction equations. However, in this study, the target strength was limited. Thus, the predicted strengths of NC and LC are limited to the range from 30 to 60 MPa. That is, target strengths under 30 MPa and above 60 MPa were not included in the experiments. Therefore, additional experiments should be conducted setting broader target-strength ranges. In addition, mechanical characteristics were measured in this study only up until 28 days of age. Therefore, the proposed equations can be applied to NC and LC up until they are 28 days old, while experiments on NC and LC after 28 days are required to predict strength at long-term ages. Future research will be focused on stress–strain relationships and microstructural analyses. These analyses are beyond the scope of this study.

## Figures and Tables

**Table 1:** Previous study for developing prediction equations.

Researcher	Prediction Equation
Lee [21]	Fc = 0.0184 × e^1.78 Vp^
J. Pyszniak [22]	Fc = (92.5 Vp^2^ − 508 Vp + 782)/10.2
AIJ [23]	Fc = (215 Vp − 620)/10.2
Kim [24]	Fc = 33.53 Vp − 103.38

(1) Fc: compressive strength (MPa); (2) Vp: ultrasonic pulse velocity (km/s).

**Table 2:** Physical properties of the materials.

Materials	Properties
Cement	Type I ordinary Portland cement Density: 3150 kg/m^3^, fineness: 320 m^2^/kg
Coarse aggregate	Crushed granite aggregate Density: 2680 kg/m^3^, fineness modulus: 7.03 Absorption: 0.68%, maximum size: 20 mm
	Coal ash aggregate Density: 1470 kg/m^3^, fineness modulus: 6.39 Absorption: 8.68%, maximum size: 20 mm
Fine aggregate	River sand Density: 2540 kg/m^3^, fineness modulus: 2.54 absorption: 1.6%
Super plasticizer	Polycarboxylic-based acid

**Table 3:** Chemical properties of cement.

Materials	Chemical Composition (%)								L.O.I
	CaO	SiO_2_	Al_2_O_3_	Fe_2_O_3_	MgO	SO_3_	K_2_O	Others	
OPC	60.3	19.8	4.9	3.3	3.8	2.9	1.1	0.9	3

(1) OPC: ordinary Portland Cement; (2) L.O.I: loss on ignition.

**Table 4:** Experimental program.

Classification	Tests Conditions
Specimen dimension	Φ100 × 200 mm
Type of coarse aggregate	Crushed granite aggregate, Coal ash aggregate
Purpose compressive strength	30, 45, and 60 MPa
Curing conditions	Room temperature: 20 ± 2 °C, Humidity: 60 ± 5%
Test items	Compressive strength (MPa), ultrasonic pulse velocity (km/s), Elastic modulus (GPa)

**Table 5:** Mix proportions of the NC and LC.

MIX ID	f_ck_ (MPa)	W/B	S/a (%)	Unit Weight (kg/m^3^)			
				W	C	S	G
LC30	30	0.41	46.0	165	400	799	758
NC30					400	799	956
LC45	45	0.33	43.0		500	711	762
NC45					500	711	961
LC60	60	0.28	43.0		600	676	724
NC60					600	676	913

(1) W/B: water/binder; (2) S/a: sand/aggregate; (3) W: water; (4) C: cement; (5) S: sand; (6) G: gravel.

**Table 6:** Testing methods used for determining the mechanical properties.

Test Items	Test Method	Test Ages
Compressive strength (MPa)	ASTM C39/C39M [26]	1, 3, 7, 28 day
Ultrasonic pulse velocity (km/s)	ASTM C597 [27]	
Elastic modulus (GPa)	ASTM C469 [28]	3, 7, 28 day

**Table 7:** Correlation between the compressive strength and the ultrasonic pulse velocity.

ID	Equation	Correlation Coefficient (R^2^)
NC30	Fc = 0.1945 × e^1.2372Vp^	R^2^ = 0.79
LC30	Fc = 0.1251 × e^1.5176Vp^	R^2^ = 0.97
NC45	Fc = 0.8276 × e^0.8764Vp^	R^2^ = 0.98
LC45	Fc = 0.432 × e^1.1755Vp^	R^2^ = 0.92
NC60	Fc = 2.0175 × e^0.7549Vp^	R^2^ = 0.93
LC60	Fc = 0.9628 × e^0.9576Vp^	R^2^ = 0.65

**Table 8:** Correlation between the elastic modulus and the ultrasonic pulse velocity.

ID	Equation	Correlation Coefficient(R^2^)
NC30	E = 0.3837 × e^0.0057Vp^	R^2^ = 0.91
LC30	E = 1.1907 × e^0.7565Vp^	R^2^ = 0.98
NC45	E = 3.3527 × e^0.4648Vp^	R^2^ = 0.91
LC45	E = 6.459 × e^0.3106Vp^	R^2^ = 0.75
NC60	E = 2.0175 × e^0.7549Vp^	R^2^ = 0.93
LC60	E = 4.4592 × e^0.3964Vp^	R^2^ = 0.84

## Data Availability

The data presented in this study are available on request from the corresponding author.
